# The Impact of Immunosenescence on Humoral Immune Response Variation after Influenza A/H1N1 Vaccination in Older Subjects

**DOI:** 10.1371/journal.pone.0122282

**Published:** 2015-03-27

**Authors:** Iana H. Haralambieva, Scott D. Painter, Richard B. Kennedy, Inna G. Ovsyannikova, Nathaniel D. Lambert, Krista M. Goergen, Ann L. Oberg, Gregory A. Poland

**Affiliations:** 1 Mayo Vaccine Research Group, Mayo Clinic, Rochester, Minnesota, United States of America; 2 Program in Translational Immunovirology and Biodefense, Mayo Clinic, Rochester, Minnesota, United States of America; 3 Division of Biomedical Statistics and Informatics - Department of Health Science Research, Mayo Clinic, Rochester, Minnesota, United States of America; 4 Department of Pediatric and Adolescent Medicine, Mayo Clinic, Rochester, Minnesota, United States of America; Deakin University, AUSTRALIA

## Abstract

**Background:**

Although influenza causes significant morbidity and mortality in the elderly, the factors underlying the reduced vaccine immunogenicity and efficacy in this age group are not completely understood. Age and immunosenescence factors, and their impact on humoral immunity after influenza vaccination, are of growing interest for the development of better vaccines for the elderly.

**Methods:**

We assessed associations between age and immunosenescence markers (T cell receptor rearrangement excision circles – TREC content, peripheral white blood cell telomerase – TERT expression and CD28 expression on T cells) and influenza A/H1N1 vaccine-induced measures of humoral immunity in 106 older subjects at baseline and three timepoints post-vaccination.

**Results:**

TERT activity (TERT mRNA expression) was significantly positively correlated with the observed increase in the influenza-specific memory B cell ELISPOT response at Day 28 compared to baseline (p-value=0.025). TREC levels were positively correlated with the baseline and early (Day 3) influenza A/H1N1-specific memory B cell ELISPOT response (p-value=0.042 and p-value=0.035, respectively). The expression and/or expression change of CD28 on CD4+ and/or CD8+ T cells at baseline and Day 3 was positively correlated with the influenza A/H1N1-specific memory B cell ELISPOT response at baseline, Day 28 and Day 75 post-vaccination. In a multivariable analysis, the peak antibody response (HAI and/or VNA at Day 28) was negatively associated with age, the percentage of CD8+CD28low T cells, IgD+CD27- naïve B cells, and percentage overall CD20- B cells and plasmablasts, measured at Day 3 post-vaccination. The early change in influenza-specific memory B cell ELISPOT response was positively correlated with the observed increase in influenza A/H1N1-specific HAI antibodies at Day 28 and Day 75 relative to baseline (p-value=0.007 and p-value=0.005, respectively).

**Conclusion:**

Our data suggest that influenza-specific humoral immunity is significantly influenced by age, and that specific markers of immunosenescence (e.g., the baseline/early expression of CD28 on CD4+ and/or CD8+ T cells and T cell immune abnormalities) are correlated with different humoral immune response outcomes observed after vaccination in older individuals, and thus can be potentially used to predict vaccine immunogenicity.

## Introduction

Influenza vaccination continues to be an important method to protect against influenza and influenza-related complications [1,2,3]. However, influenza vaccines have reduced immunogenicity and efficacy in the elderly, and age-related alterations of the immune system are known to affect immune responses following influenza vaccination [4,5,6,7]. Despite annual vaccine coverage, more than 90% of the 36,000 influenza-related annual deaths occur in adults 65 years of age and older [1]. In order to develop more efficient approaches for protection against influenza in the elderly, immunosenescence and vaccine-induced immune responses require greater comprehension, including understanding the immune response dynamics and correlates of protection following immunization, as well as the interrelationships and dependencies among various immune response variables that determine and/or perturb immune function.

Previous reports from the literature, including our own, suggest the importance of age and specific markers of immunosenescence (e.g., CD28 expression on T cells, the expression levels of the peripheral white blood cell telomerase TERT, Th1/Th2 cytokine disbalance, etc.) for diminished vaccine-induced immune responses in older and elderly individuals [6,7,8,9,10]. Recent animal studies provide quantitative analyses and modeling of immune components during influenza infection in young and aged mice and demonstrate the key role of CD8+T cells and cytokines (IFNα/β, IFNγ and TNFα) for viral clearance [11]. However, age and immunosenescence have not been systematically studied in regard to influenza vaccination in humans–particularly their influence on the magnitude and kinetics of various humoral immune response variables. Such data could fill the knowledge gap and aid the development of vaccines with higher immunogenicity and efficacy in the elderly.

The humoral branch of adaptive immunity responds to vaccination/infection by activating and differentiating antigen-specific B cells to produce influenza-specific antibodies that neutralize and/or clear the influenza virus by cell-dependent mechanisms (e.g., antibody-dependent cellular cytotoxicity [12]). During the course of humoral immune response, antigen-specific B cells (including peripheral B cell subsets such as antibody-secreting cells and memory B cells) and antibodies are known to peak at specific timepoints after exposure to influenza virus antigens [13,14]. Currently, correlates of protection for influenza-specific humoral immunity are primarily based on assessment/quantification of antibodies by the hemagglutination inhibition (HAI) and virus neutralization (VNA) assays. Seroprotection against influenza is defined as a HAI titer of 1:40 or greater [15]. However, alternative correlates of immunity (e.g., measures of cellular immunity, antigen-specific and total peripheral B cell immune responses) are warranted for in-depth evaluation of immune preparedness in older individuals [16,17]. More comprehensive assessment of humoral immune response variations with respect to age and immunosenescence in older adults could provide additional data on the impact of age-related immunological defects on humoral immune response following influenza vaccination.

We hypothesized that age and specific quantitative markers of immunosenescence are associated with influenza-specific and overall inter-individual immune response variations after vaccination in older individuals. We studied a cohort of older subjects (n = 106) at selected timepoints pre- and post-vaccination and identified associations between the expression of CD28 on CD8+ and/or CD4+ T cells (as well as other flow cytometry/immunosenescence variables) and the observed peak humoral immune response outcomes (HAI, VNA and memory B cell ELSPOT) after vaccination. This could provide valuable information regarding the immunogenicity and efficacy of influenza vaccines in this age group.

## Methods

The methods described herein are similar or identical to those published for our previous studies [9,18,19].

### Study subjects

As previously reported, the sample population consisted of 106 healthy subjects (50 to 74 years old) from Olmsted County, MN [18,19]. The 2010–2011 licensed trivalent influenza vaccine, containing the A/California/7/2009 H1N1-like, A/Perth/16/2009 H3N2-like, and B/Brisbane/60/2008-like viral strains, was administered to all study participants. Blood samples were collected once prior to vaccination (baseline) and at three timepoints post-vaccination (Day 3, Day 28, and Day 75), yielding a total volume of approximately 400 milliliters of blood per subject.

All study participants provided written informed consent, and all study procedures were approved by the Institutional Review Board of the Mayo Clinic.

### Isolation of peripheral blood mononuclear cells (PBMCs)

Peripheral blood mononuclear cells (PBMCs) were isolated from each subject at each timepoint post-vaccination (Day 0, 3, 28 and 75) from 100 mL of whole blood using Cell Preparation Tube with Sodium Citrate (CPT) tubes as previously described [20]. Cells were re-suspended at a concentration of 1×107/mL in RPMI 1640 medium containing L-Glutamine (Invitrogen, Carlsbad, CA) supplemented with 10% dimethyl sulfoxide (DMSO; Protide Pharmaceuticals, St. Paul, MN) and 20% fetal calf serum (FCS; Hyclone, Logan, UT), frozen overnight at − 80°C in Thermo Scientific freezing containers (Thermo Fisher Scientific, Waltham, MA) to achieve an optimal rate of cooling, and then transferred to liquid nitrogen for storage, as previously reported [20].

### Growth of influenza virus

The influenza A/California/7/2009/H1N1-like strain was obtained from the Centers for Disease Control and Prevention (CDC, Atlanta, GA). The virus was grown on embryonated chicken eggs, and the allantoic fluid containing the virus was harvested and titered by hemagglutination (HA) and 50% Tissue Culture Infectious Doses (TCID50) assay following Madin-Darby canine kidney epithelial cells (MDCK) infection using standard protocols [18,19].

### Hemagglutination Inhibition (HAI) Assay

Influenza A/California/7/2009 (H1N1)-specific HAI titers were measured in subjects’ sera at each timepoint pre- and post-vaccination (Day 0, 3, 28 and 75) using a standard protocol, as described elsewhere [17–19]. Briefly, to eliminate non-specific inhibitors of hemagglutination, all sera were first treated with receptor-destroying enzyme (RDE, *Vibrio cholerae* filtrate, Sigma-Aldrich, St. Louis, MO), as previously reported [21]. Serial two-fold dilutions of the pretreated and inactivated sera (25 μl) were mixed with 4 hemagglutination (HA) units/25 μl of influenza virus and incubated for 30 minutes at room temperature to allow antigen-antibody binding. An equal volume (50 μl) of 0.5% turkey red blood cell suspension was then added to the mixture. HAI titers were determined after a 45-minute incubation on ice as the reciprocal of the highest serum dilution that completely inhibits hemagglutination [18].

### Viral neutralization assay

Influenza A/H1N1-specific neutralizing antibody titers were determined through a cell-based virus neutralization assay using subject sera collected at each timepoint pre- and post-vaccination (Day 0, 3, 28, 75). In brief, MDCK (Madin-Darby canine kidney) cells were placed in flat-bottom 96-well plates (10^4^ cells/well) and incubated at 37°C until confluent. The sera of patients were treated with RDE of *Vibrio cholerae* filtrate (Sigma-Aldrich, St. Louis, MO) and incubated overnight at 37°C to eliminate nonspecific inhibitors. The next day, 2.5% sodium citrate solution was added to the RDE-treated sera and heated at 56°C for 30 minutes. Starting dilution (1:10) was adjusted by adding Phosphate Buffer Solution (PBS, pH 7.3). The RDE-treated sera (25 μl) were then added to the first well of a V-bottom 96-well plate and diluted with PBS (25 μl). Serial two-fold dilutions were performed in each row by transferring 25 μl of diluted sera to each consecutive well containing 25 μl PBS. This resulted in 12 dilutions from 1:2 to 1:4,096. Sera dilutions were incubated with the influenza A/H1N1 virus (200 plaque forming units/PFUs per 5 μl) for 30 minutes at room temperature to allow binding/neutralization. After incubation, the influenza virus/RDE-treated serum mixtures (30 μl) were transferred from the V-bottom 96-well plate to the corresponding wells in the flat-bottom 96-well plate containing the MDCK cells. These plates were incubated at 37°C for 60 minutes to allow viral binding/entry into the MDCK cells, after which the inocula (influenza virus/serum mixtures) were removed, the cell monolayers were gently rinsed with PBS, and Opti-MEM supplemented with Penicillin-Streptomycin and 1 μg/mL trypsin (Trypsin from bovine pancreas-TPCK-treated, Sigma-Aldrich, St. Louis, MO) was added to each well. Plates were further incubated at 37°C for four days. Viral infection was assessed by performing a hemagglutination assay on cell supernatants with 0.5% turkey red blood cell suspension as previously described [22]. The neutralization titer was determined by the last well/dilution with complete viral neutralization and no hemagglutination observed (RBCs form a button). The reciprocal value of the corresponding serum dilution multiplied by a factor of 10 (to correct for the 1:10 dilution of the serum as a result of the RDE treatment) was the recorded neutralization titer.

### B cell ELISPOT assay

We quantified influenza virus-positive B cells (memory-like IgG B cells) in subjects’ PBMCs using the Mabtech ELIspot^PLUS^ kit for human IgG (Mebtech Inc., Cincinnati, OH) according to the manufacturer’s specifications, and as previously described [19,23,24]. In summary, 96-well Millipore Immobilon-P-Membrane multiscreen filter (PVDF) plates (EMD Millipore, Billerica, MA) were pre-coated with whole influenza A/H1N1 virus at a dilution 1/10 (50,000 TCID_50_ per well) in phosphate buffered saline (PBS, pH 7.4) or with 15 μg/mL of anti-human total IgG capture mAbs MT91/145 (1.5μg per well) and incubated overnight at 4°C. For each subject, one control well was coated with PBS, pH 7.4, to represent a subject-specific background measure (negative control). In addition, for each subject we quantified the influenza virus-specific response in quadruplicate and the total IgG response (a positive control) in triplicate. Cryopreserved PBMCs were thawed as previously reported [20], extensively washed, counted and pre-stimulated in 24-well plates (4 x 10^6^ cells/well) with human recombinant IL-2 (10 ng/mL final concentration) and R848 (a TLR7/8 agonist at 1 μg/mL final concentration) at 37°C in a 5% CO2 humidified incubator for 72 hours. Before assay setup, ELISPOT plates were washed (5x with sterile PBS, pH 7.4) and blocked for 1 hour at room temperature with RPMI medium containing 10% FCS. Pre-stimulated PBMCs were collected, washed to ensure removal of any secreted antibodies, counted and plated in the antigen-coated ELISPOT plates at 2×10^5^ cells/well (for the influenza virus-specific response and the negative control), or at 1×10^4^ cells/well (for the total IgG response, positive control) in RPMI medium containing 5% FCS. Plates were then incubated for 20 hours at 37°C in 5% CO_2_. After the incubation period, the plates were washed (5x with PBS, pH 7.4) and incubated with a detection biotinylated anti-human IgG mAbs MT78/145 (at 1 μg/mL in PBS-0.5% FCS) for 2 hours at room temperature, following the manufacturer’s specifications. After washing, the plates were further incubated (1 hour, room temperature) with a streptavidin-horseradish peroxidase/HRP conjugate (dilution 1:1,000) and developed until distinct spots emerged (5 minutes, room temperature, in the dark) using a tetramethylbenzidine (TMB) peroxidase substrate solution according to the manufacturer’s protocol. Plates were scanned and analyzed using pre-optimized counting parameters on an automated ImmunoSpot S6Macro696 Analyzer (Cellular Technology Ltd., Cleveland, OH, USA) with the ImmunoSpot version 5.1 software (Cellular Technology Ltd.). Quality control (QC) was performed by a single operator to ensure QC and consistent results [20]. The results are presented in spot-forming units (SFUs) per 2×10^5^ cells as subjects’ medians (median of influenza virus-specific response, measured in quadruplicate).

### T Cell Receptor Rearrangement Excision Circles (TREC) analysis

Signal joint (sj) TREC content was assessed relative to CCR5 copies in genomic DNA (50 to 400 ng input) isolated from PBMCs. Quantitative real-time PCR for sjTREC with specific primers (forward CACATCCCTTTCAACCATGCT and reverse GCCAGCTGCAGGGTTTAGG), Taqman probe, genomic DNA template and optimized PCR conditions in a spectrofluorometric thermal cycler (ABI PRISM 7900, PE Applied Biosystems, Foster City, CA) were used, as previously described in detail [25]. Standard curves with 1–100,000 copies of human sjTREC plasmid and human CCR5 plasmid were generated (for each plate) in order to calculate copies of TREC versus CCR5 copies (ratio) in genomic DNA for each sample.

### Peripheral white blood cell (WBC) telomerase (TERT) analysis

Expression of TERT mRNA was measured in total mRNA extracted from cryopreserved PBMCs, using quantitative real-time PCR assay, as previously described with minor modifications [9]. Reference control TERT and GAPDH transcripts were generated from MCF-7 cell line by PCR, cloning into TOPO II cloning vector (Invitrogen Corp.) and production of RNA transcripts using linearized DNA templates with the RiboMAX Large Scale RNA Production System (Promega) following the manufacturer’s protocol. The reference TERT and GAPDH controls were assayed simultaneously with samples. RNA from samples was reverse transcribed to cDNA and was further assessed in duplicate via quantitative real-time PCR using TaqMan chemistry (ABI, Foster City, CA, USA), instrumentation (ABI 7900HT sequence Detection System), and primer/probe sets (ABI): Hs00972656_m1 ABI set for TERT and Hs99999905_m1 ABI set for GAPDH, as described previously [9]. The relative abundance of the experimental transcripts was calculated by back fitting the experimental CT values to the respective TERT or GAPDH standard curve produced by the reference sample titration. The results were normalized and reported as the TERT expression relative GAPDH expression (ratio) by dividing the relative abundance value for TERT by the abundance value for GAPDH.

### Multicolor flow cytometry

Frozen PBMCs from all collected pre- and post-vaccination timepoints were thawed and subjected to standard staining with fluorescent antibody conjugates, data acquisition and data analysis in two multicolor panels: B-cell specific panel; and CD4/CD8 T cell-specific panel for CD28 expression. The B-cell panel consisted of a cocktail to measure the following markers using commercially available labeled antibodies CD3 (Cat. #55783, BD Biosciences; San Jose, CA), CD19 (Cat. #561121, BD Biosciences; San Jose, CA), CD20 (Cat. #562873, BD Biosciences; San Jose, CA), CD24 (Cat. #561646, BD Biosciences; San Jose, CA), CD27 (Cat. #558664, BD Biosciences; San Jose, CA), CD38 (Cat. #562665, BD Biosciences; San Jose, CA), and IgD (Cat. #555778, BD Biosciences; San Jose, CA). The T-cell panel consisted of antibodies to measure CD3 (Cat. #55783, BD Biosciences; San Jose, CA), CD4 (Cat. #557922, BD Biosciences; San Jose, CA), CD8 (Cat. #562428, BD Biosciences; San Jose, CA), and CD28 (Cat. #562613, BD Biosciences; San Jose, CA) expression. Samples were analyzed using a BD LSR II Flow Cytometer. Cell population gating was performed using FlowJo v10 (Treestar; Ashland, OR).

### Statistical analysis

Histograms and box plots by assay batch were used to assess data quality. Due to skewness in the distributions, nonparametric methods were used for univariable hypothesis testing. Thus, data are summarized via quantiles. Nonparametric Wilcoxon sign rank tests or Friedman rank sum (a non-parametric repeated measures test) tests were applied to test for differences. Spearman’s nonparametric correlation was used to assess correlations between measures. The significance criterion was p-value<0.05. The Day 3 immunosenescence/flow cytometry variables were included in a multivariable analysis to assess early “predictors”/variables associated with the observed peak influenza vaccine-specific humoral immune response outcomes (HAI, VNA, B cell ELISPOT at Day 28). Elastic net penalized linear regression[26], which accommodates highly correlated predictor variables with α = 0.9, was used with an Analysis of Covariance strategy. Specifically, the dependent variable was the Day 28 HAI/VNA/B cell ELISPOT value (on the log_2_ scale), and both the Day 0 value (also on the log_2_ scale) and gender were forced in the model as covariates or independent predictor variables. The Day 3 immunosenescence variables, flow cytometry variables, and age were then allowed to compete for entry into the model as potential independent predictors. This was done separately for HAI, VNA and B cell ELISPOT. Models were fit using the “glmnet” function in R [27]. Models with minimum mean squared error were selected using 10-fold cross validation [27].

## Results

### Study Subjects

As previously reported [18,19], the study cohort consisted of 106 generally healthy older adults (50–74 years old): 65 (61.3%) females and 41 (38.7%) males. The median age for the study cohort was 59.7 years (IQR 55.3, 67.6). One-hundred-four subjects (98.1%) were Caucasian, and two of the subjects were of other races (i.e., one subject was Asian and one was multi-race).

### Major Humoral Immune Response Variables

**Fig 1:**
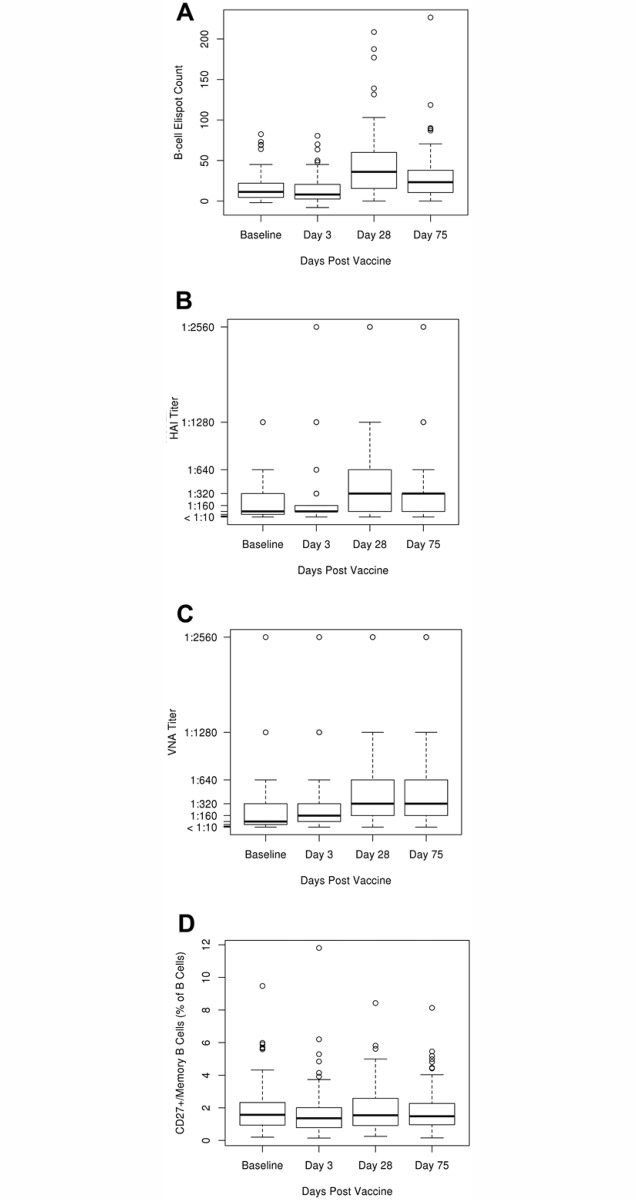
Box and whisker plots of major humoral immune response outcomes pre-vaccination (baseline) and post-vaccination (Day 3, Day 28, and Day 75). The top (bottom) of the box indicate the 75^th^ (25^th^) percentiles, respectively, while the bold line within the box indicates the median. The ‘whiskers’ extend up to 1.5 times the interquartile range above or below the 75^th^ or 25^th^ percentiles respectively. Beyond that point, individual points are plotted. **A.** B cell ELISPOT counts representing the influenza A/H1N1-specific IgG-producing memory-like B cell response plotted for each timepoint as spot forming units (SFUs) per 2x10^5^ PBMCs. **B.** HAI antibody titers (presented as the inverse of the greatest serum dilution that still gave a positive HAI result) are plotted over time. **C.** Viral neutralization antibody (VNA) titers (presented as the inverse of the greatest serum dilution that still gave a positive VN result) are plotted over time. **D.** CD20+ CD27+memory B cells (% of B cells) over time. Parts of Fig. 1(Fig. 1A) have been published in Viral Immunology [18,19].

The humoral immune response variables pre- and post-influenza vaccination are summarized in S1 Table and Fig. 1. The median HAI titer at Day 28 post-vaccination was 320 (presented as the inverse of the greatest serum dilution that still gave a positive result; IQR, 80, 640) with 37 subjects (38.95%, 37 out of 95, excluding 11 subjects with baseline glass ceiling titers of>640) having ≥4-fold increase in HAI titer from Day 0 to Day 28 post-vaccination. The median viral neutralization antibody (VNA) titer at Day 28 post-vaccination was 320 (presented as the inverse of the greatest serum dilution that still gave a positive result; IQR 160, 640) with 45 subjects (47.37%, 45 out of 95) having ≥4-fold increase in VNA titer from Day 0 to Day 28 post-vaccination. We were unable to detect any measurable response in the B cell ELISPOT assay, measuring influenza A/H1N1-specific antibody-secreting cells (ASCs)/plasmablasts at all specific pre- (Day 0) and post-vaccination timepoints (Day 3, Day 28 and Day 75; data not shown). The median influenza A/H1N1-specific B cell ELISPOT response (as represented by the influenza A/H1N1-specific IgG-producing memory-like B cell counts) was 11 SFUs per 2x10^5^ PBMCs (IQR 5, 22) at baseline, dropped down to 8 SFUs per 2x10^5^ PBMCs (IQR 3, 20) at Day 3 post-vaccination, and then increased to 36 SFUs (IQR 16, 60), and 23 SFUs (IQR 11, 38) at Day 28 and Day 75 (respectively) post-influenza vaccination (S1 Table, Fig. 1) [19].

Forty-three subjects (40.6%) had a ≥4-fold increase in influenza A/H1N1-specific B cell ELISPOT response (influenza A/H1N1-specific memory-like B cell counts) from Day 0 to Day 28 post-vaccination. The overall CD20+CD27+ memory B cell frequencies (switched and unswitched) (measured by flow cytometry as % of B cells) were 1.57% pre-vaccination (IQR 0.94, 2.33), dropped down to 1.36% (IQR 0.78, 2.01) at Day 3 post-vaccination and gradually returned back to baseline values at Day 28 (1.54%) and Day 75 (1.48%) post-influenza vaccination (S1 Table, Fig. 1). The overall IgD-CD27- memory B cell frequencies (measured by flow cytometry as % of B cells) were 1.08% (IQR 0.65, 1.68) pre-vaccination, and dropped down to 0.8% measured at Day 3, Day 28 and Day 75 post-vaccination (S1 Table). The overall CD20-CD27^high^CD38^high^ plasma cells/plasmablasts were 0.15–0.16% (as % of B cells) at all timepoints (S1 Table). No statistically significant sex-related differences were observed for the influenza virus-specific immune outcomes, although the results suggest females have higher frequencies of influenza A/H1N1-specific IgG-producing memory-like B cells (influenza-specific IgG memory-like B cell ELISPOT response) at all pre-and post-vaccination timepoints compared to males (S2 Table).

### Timepoint Kinetics of Humoral Immune Response Relative to Influenza Vaccination

To assess the dynamics of humoral immune response following influenza vaccination, we also compared the median value for each humoral immune response variable between all pre- and post-vaccination timepoints, using a Wilcoxon signed rank test and a Friedman rank sum test for the overall comparisons (S3 Table, Fig. 1). As expected, there were statistically significant changes (increase) in influenza A/H1N1-specific serum HAI titers at later points after influenza vaccination (Day 28 and Day 75) compared to baseline and/or Day 3 levels (overall p-value = 1.6E^-28^). Similarly, we observed a significant increase in VNA titers at all post-vaccination timepoints relative to baseline (overall p-value = 8.4E^-32^). As noted above, we also observed statistically significant changes in influenza A/H1N1-specific B cell ELISPOT response measures over time (overall p-value = 5.8E^-26^), characterized by lower measures at Day 3 relative to baseline (p = 0.001) and a significant increase at Day 28 (p = 1.5E^-13^) and Day 75 (p = 6.3E^-08^) relative to pre-vaccination levels (S3 Table, Fig. 1). A similar drop at Day 3 (relative to baseline) was observed in the overall CD20+CD27+ memory B cell frequencies (measured by flow cytometry), with a gradual return to the pre-vaccination measures at Day 28 and Day 75 post-vaccination (S1 and S3 Tables). Other cell populations/flow cytometry variables also displayed significant differences over time relative to vaccination (S3 Table). No significant changes in the overall CD20-CD27^high^CD38^high^ plasma cells/plasmablasts frequencies were noted over time (overall p-value = 0.53), but we observed a slight increase of the overall frequencies of CD20-/CD27+CD38+ plasma cells/% of B cells at Day 3 relative to baseline (Day 0) (p-value = 0.006, S3 Table), and Day 75 relative to baseline (p-value = 0.007, S3 Table).

### Associations of Age and Immunosenescence with Humoral Immunity and Vaccine Response

**Fig 2:**
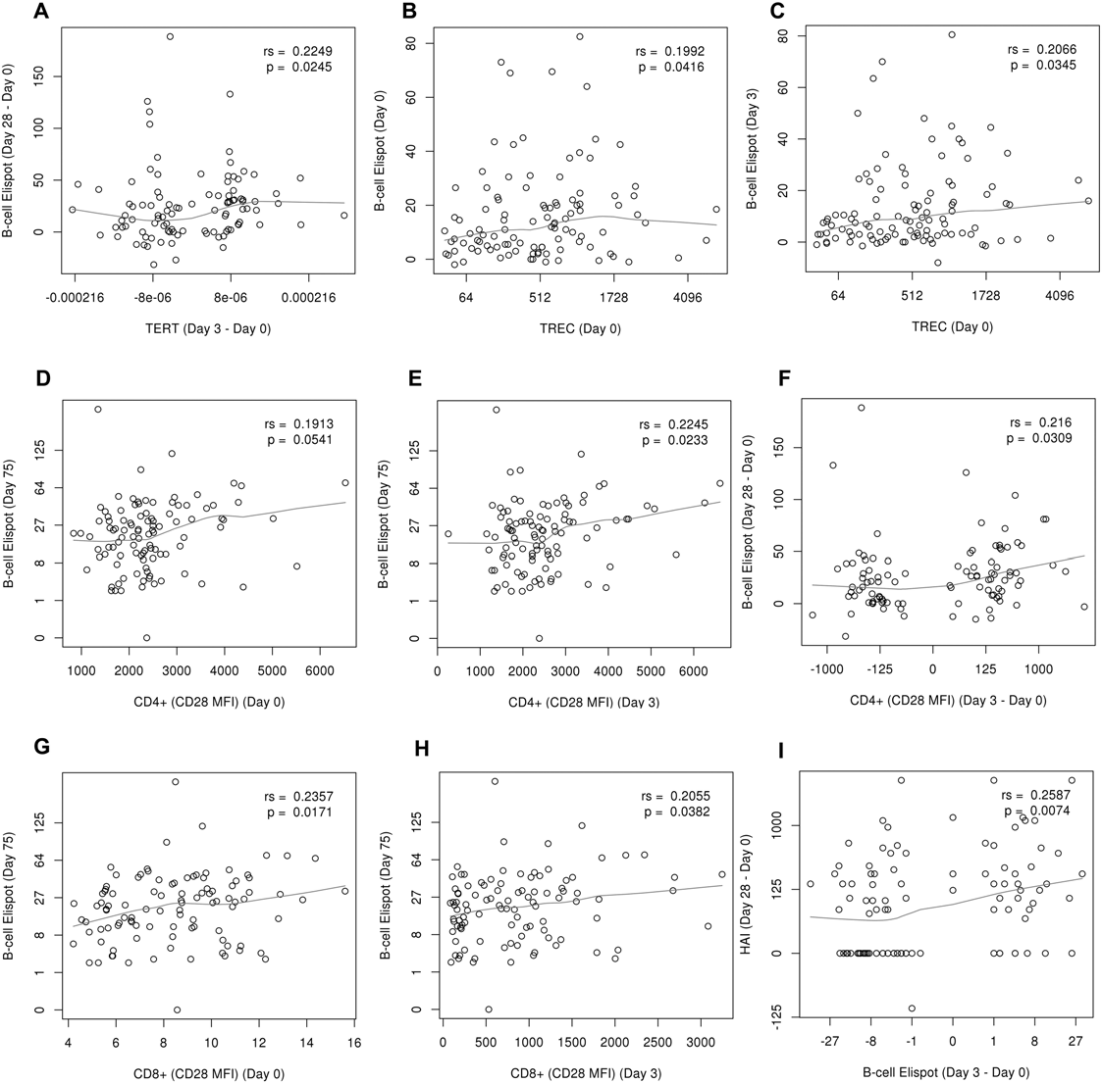
Correlations between markers of immunosenescence (and/or immune outcomes) and influenza A/H1N1 vaccine-induced immune responses. **A.** The panel illustrates the positive correlation between early change in TERT expression at Day 3 relative to baseline on x-axis and influenza-specific B cell ELISPOT response change on y-axis (Day 28 compared to baseline). **B and C.** The panels illustrate the positive correlation between TREC levels on x-axis and influenza-specific B cell ELISPOT response on y-axis (Day 0 or Day 3). **D**, **E** and **F.** The panels illustrate the positive correlation between CD28 expression (MFI) on CD4+ T cells on x-axis (or change in expression at Day 3 relative to baseline, panel F) and influenza-specific B cell ELISPOT response on y-axis (Day 75 or increase at Day 28 compared to baseline for panel F). **G** and **H.** The panels illustrate the positive correlation between CD28 expression (MFI) on CD8+ T cells on x-axis (Day 0 or Day 3) and influenza-specific B cell ELISPOT response at Day 75 on y-axis. **I.** The panel illustrates the positive correlation between early change in influenza-specific B cell ELISPOT response (Day 3 relative to baseline, on x-axis) and influenza-specific HAI titer increase (Day 28 compared to baseline, on y-axis). The values on the x-axis for panels A, B, C, and F, y-axis for panels D, E, G, and H, and both x- and y-axes for panel I are displayed on the cube root scale for ease of visualization, but labeled on the raw scale. Influenza A/H1N1-specific memory-like IgG B cell ELISPOT response is presented as median SFUs per 2 x 10^5^ cells; TERT expression is presented as TERT relative abundance value (relative expression in mRNA) divided by the GAPDH abundance value (ratio); TREC levels are presented as TREC versus CCR5 copies (ratio) in genomic DNA; HAI represents the influenza A/H1N1-specific hemagglutination inhibition titer as the reciprocal of the highest serum dilution that completely inhibits hemagglutination. The expression of CD28 on CD4+ and/or CD8+ T cells is measured by the mean fluorescence intensity/MFI using flow cytometry. “rs” indicates Spearman’s correlation coefficient.

We assessed the correlations between influenza vaccine-specific humoral immunity measures and other overall humoral immune response variables measured over time, and age/immunosenescent markers (TREC, TERT, CD28 expression on CD4+ and CD8+ T cells) (Table 1, Fig. 2). The HAI and neutralization titers at Day 28 following influenza vaccination were inversely correlated with age (r = -0.2, p-value = 0.04 and r = -0.19, p-value = 0.05, for HAI and VNA, respectively, Table 1). The activity of the peripheral white blood cell (WBC) telomerase TERT (as assessed by the expression of TERT mRNA at different timepoints relative to vaccination), a measure linked to telomere length maintenance and inversely linked to replicative senescence, was positively associated with HAI and neutralization antibody levels measured at Day 3 post-vaccination. The early change in the TERT expression (Day 3 compared to baseline) was positively correlated with the observed increase in the influenza-specific B cell ELISPOT response at Day 28 post-vaccination compared to baseline (r = 0.225, p-value = 0.025, Table 1). In addition, TERT levels were positively correlated with the overall frequencies of several B cell and plasma cell populations at different timepoints post-vaccination (e.g., CD20-/CD27high plasma cells/% of B cells at Day 0 and Day 3; CD20+/CD27+ B cells/% of B cells at Day 28, Table 1). The TREC levels (by-products of the DNA rearrangements during the process of TCR recombination, and an estimate of thymic function and immunosenescence) were positively correlated with the baseline (Day 0) and early (Day 3) influenza A/H1N1-specific B cell ELISPOT response (p-value = 0.04 and p-value = 0.035, respectively, Table 1). In addition, we observed a negative correlation between TREC levels and the overall IgD-CD27- B cell frequencies (% of B cells), as well as the overall IgD-CD27-memory B cell frequencies (% of B cells) at Day 28 following vaccination (p-value = 0.025 and p = 0.008, respectively, Table 1). The expression of CD28 on CD4+ and CD8+ T cells (as measured by the mean fluorescence intensity/MFI using flow cytometry) at baseline and Day 3 was positively correlated with the influenza A/H1N1-specific B cell ELISPOT response at baseline and Day 75 (p≤0.05, Table 1). In addition, the early change/increase of CD4+ T cell CD28 expression at Day 3 (relative to baseline) was significantly correlated with the observed increase in the influenza-specific B cell ELISPOT response at Day 28 post-vaccination compared to baseline (r = 0.216, p-value = 0.031, Table 1). Consistent with these findings, our univariable analysis demonstrated an inverse correlation (r = -0.186) between the frequencies of CD8+ T cells with low and/or negative CD28 expression (as % of CD8+ T cells) and influenza A/H1N1-specific B cell ELISPOT response at Day 75 that did not quite reach statistical significance (p = 0.06, data not shown).

**Table 1:** Influence of age and immunosenescence on immune response variables after influenza A/H1N1vaccination.

Variable	Immune outcome	Correlation coefficient^a^	P-value^b^
Age	HAI—Day 28	-0.200	0.040
Age	VNA—Day 28	-0.190	0.05
Age	CD27+/Memory B Cells (% of B Cells)—Day 3	0.220	0.036
Age	IgD+CD27-/Transitional B Cells (% of B Cells)—Day 28	-0.216	0.037
Age	IgD-CD27-/Transitional B Cells (% of B Cells)—Day 28	-0.260	0.012
TERT—Day 3	HAI—Day 3	0.224	0.024
TERT—Day 3	VNA—Day 3	0.207	0.037
TERT—Day 28	CD20+/CD27+ B Cells (% of B Cells)—Day 28	-0.230	0.029
TERT—Day 28	CD27+/Memory B Cells (% of B Cells)—Day 28	-0.218	0.038
TERT—Day 0	CD20-/CD27high Plasma Cells (% of B Cells)—Day 0	0.215	0.04
TERT—Day 3	CD20-/CD27high Plasma Cells (% of B Cells)—Day 3	0.253	0.018
TERT—(Day 3—Day 0)	B-Cell ELISPOT^c^ –(Day 28—Day 0)	0.225	0.025
TREC—Day 0	B-Cell ELISPOT—Day 0	0.199	0.042
TREC—Day 0	B-Cell ELISPOT—Day 3	0.207	0.035
TREC—Day 0	IgD-CD27- B Cells (% of B Cells)—Day 28	-0.233	0.025
TREC—Day 0	IgD-CD27-/Memory B Cells (% of B Cells)—Day 28	-0.273	0.008
CD4+ (CD28 MFI)—Day 0	B-Cell ELISPOT—Day 0	0.241	0.015
CD4+ (CD28 MFI)—Day 0	B-Cell ELISPOT—Day 75	0.191	0.05
CD4+ (CD28 MFI)—Day 3	B-Cell ELISPOT—Day 75	0.224	0.023
CD4+ (CD28 MFI)–(Day 3—Day 0)	B-Cell ELISPOT—(Day 28—Day 0)	0.216	0.031
CD8+ (CD28 MFI)—Day 0	B-Cell ELISPOT—Day 75	0.236	0.017
CD8+ (CD28 MFI)—Day 3	B-Cell ELISPOT—Day 75	0.206	0.038
CD8+ (CD28 MFI)–(Day 28—Day 0)	B-Cell ELISPOT—(Day 28—Day 0)	0.197	0.047

^a^Spearman’s rank r

^b^Only correlations with p-values below or equal to 0.05 are presented

^c^Influenza A/H1N1-specific memory-like IgG B cell ELISPOT response

### Correlations between Overall/Influenza-Specific Humoral Immune Response Variables and Influenza-Specific Humoral Immunity after Vaccination

To evaluate the interrelationships between humoral immune response variables relative to vaccination, we assessed correlations between observed overall and influenza-specific humoral immunity measures. The HAI antibody titers were strongly correlated with the influenza-specific neutralization (VNA) antibody titers at all timepoints pre- and post-vaccination (Table 2). Importantly, the early change in influenza-specific B cell ELISPOT response (Day 3 relative to baseline) was significantly positively correlated with the: observed increase in influenza A/H1N1-specific HAI antibodies at Day 28 post-vaccination compared to baseline (r = 0.259, p-value = 0.007, Table 2, Fig. 2); increase in influenza A/H1N1-specific HAI antibodies at Day 75 post-vaccination compared to baseline (r = 0.273, p-value = 0.005, Table 2); and increase in influenza-specific VNA antibodies at Day 75 compared to baseline (r = 0.195, p-value = 0.045, Table 2). In addition, the observed overall percentage of CD20+/CD27+ B cells (% of B cells) and CD27+/memory B cells (% of B cells) at baseline was positively correlated with the influenza-specific HAI titer measured at Day 28 and/or Day 75 post-vaccination, as well as with the observed increase of HAI titer at Day 28 compared to baseline (Day 28—Day 0 HAI titer) (Table 2). As expected, similar correlations were observed between the influenza-specific VNA antibody titers and the overall percentage of the CD20+/CD27+ and CD27+/memory B cell populations (Table 2). We also observed correlations between the influenza A/H1N1-specific B cell ELISPOT response (quantifying primarily influenza virus-specific memory-like IgG B cells) measured at different timepoints relative to vaccination, and the overall percentage of different B cell populations (Table 2). We noted consistent positive correlations between the observed influenza A/H1N1-specific B cell ELISPOT frequencies at Day 0, Day 28 and Day 75 and the overall CD27+ memory B cell frequencies (% of B cells) at Day 0, Day 28 and Day 75 relative to vaccination (e.g., overall CD27+ memory B cell counts /% of B cells at baseline were correlated with influenza A/H1N1-specific B cell ELISPOT response at Day 28, r = 0.229, p-value = 0.029, Table 2), but observed only one significant correlation between influenza-specific B cell ELISPOT response (at Day 75) and the overall percentage of plasma cells/plasmablasts at Day 75 post-vaccination (r = 0.225, p-value = 0.027, Table 2). We also observed an inverse correlation between the overall frequencies of IgD+CD27- B cells and influenza-specific B cell ELISPOT response measures at different timepoints relative to vaccination (Table 2).

**Table 2:** Correlation between overall and influenza-specific humoral immune response variables following influenza vaccination.

Variable	Influenza-specific immune outcome	Correlationcoefficient^a^	P-value^b^
VNA—Day 0	HAI—Day 0	0.942	3.04E-51
VNA—Day 3	HAI—Day 3	0.943	1.68E-51
VNA—Day 28	HAI—Day 28	0.94	1.77E-50
VNA—Day 75	HAI—Day 75	0.937	3.06E-49
B-Cell ELISPOT^c^—(Day 3—Day 0)	HAI—(Day 28—Day 0)	0.259	0.007
B-Cell ELISPOT—(Day 3—Day 0)	HAI—(Day 75—Day 0)	0.273	0.005
CD20+ B Cells (% B Cells) Day 0	HAI (Day28-Day0)	0.239	0.022
CD20+ B Cells (% B Cells) Day 3	HAI (Day3-Day0)	0.206	0.049
CD20+/CD27+ B Cells (% of B Cells)—Day 75	HAI—Day 75	0.218	0.032
CD20+/CD27+ B Cells (% of B Cells)—Day 0	HAI—Day 28	0.283	0.007
CD20+/CD27+ B Cells (% of B Cells)—Day 0	HAI—Day 75	0.248	0.018
CD27+/Memory B Cells (% of B Cells)—Day 75	HAI—Day 75	0.219	0.032
CD27+/Memory B Cells (% of B Cells)—Day 0	HAI—Day 28	0.244	0.02
CD27+/Memory B Cells (% of B Cells) Day 0	HAI (Day28-Day0)	0.207	0.049
CD27+/Transitional B Cells (% of B Cells)—(Day 3—Day 0)	HAI—(Day 3—Day 0)	0.251	0.018
IgD-CD27-/Naive B Cells (% of B Cells) Day 0	HAI—(Day 3—Day 0)	0.299	0.004
IgD-CD27-/Naive B Cells (% of B Cells) Day 3	HAI—(Day 3—Day 0)	0.280	0.007
B-Cell ELISPOT—(Day 3—Day 0)	VNA—(Day 75—Day 0)	0.195	0.045
CD20+/CD27+ B Cells (% of B Cells)—Day 0	VNA—Day 28	0.238	0.02
CD20+/CD27+ B Cells (% of B Cells)—Day 0	VNA—Day 75	0.218	0.038
CD27+/Memory B Cells (% of B Cells)—Day 0	VNA—Day 28	0.225	0.032
IgD+CD27-/Naive B Cells (% of B Cells)—Day 0	VNA—Day 75	-0.210	0.046
CD27+/Memory B Cells (% of B Cells)—Day 0	VNA—(Day 28—Day 0)	0.228	0.03
CD20+/CD27+ B Cells (% of B Cells)—Day 0	B-Cell ELISPOT—Day 0	0.294	0.005
CD20+/CD27+ B Cells (% of B Cells)—Day 0	B-Cell ELISPOT—Day 28	0.262	0.012
CD20+/CD27+ B Cells (% of B Cells)—Day 28	B-Cell ELISPOT—Day 28	0.368	0.0003
CD20+/CD27+ B Cells (% of B Cells)—Day 28	B-Cell ELISPOT—Day 75	0.308	0.003
CD20+/CD27+ B Cells (% of B Cells)—Day 75	B-Cell ELISPOT—Day 75	0.371	0.0002
CD27+/Memory B Cells (% of B Cells)—Day 0	B-Cell ELISPOT—Day 0	0.246	0.019
CD27+/Memory B Cells (% of B Cells)—Day 0	B-Cell ELISPOT—Day 28	0.229	0.029
CD27+/Memory B Cells (% of B Cells)—Day 28	B-Cell ELISPOT—Day 28	0.294	0.004
CD27+/Memory B Cells (% of B Cells)—Day 75	B-Cell ELISPOT—Day 75	0.287	0.004
CD27+/Naive B Cells (% of B Cells)—Day 3)	B-Cell ELISPOT—(Day 75—Day 0)	0.228	0.03
CD27+/Naive B Cells (% of B Cells)—Day 28	B-Cell ELISPOT—Day 28	0.293	0.004
CD27+/Naive B Cells (% of B Cells)—Day 28	B-Cell ELISPOT—(Day 28—Day 0)	0.213	0.039
CD27+/Naive B Cells (% of B Cells)—Day 28	B-Cell ELISPOT—Day 75	0.280	0.006
CD27+/Naive B Cells (% of B Cells)—Day 75	B-Cell ELISPOT—Day 75	0.367	0.0002
CD27+/Transitional B Cells (% of B Cells)—Day 0	B-Cell ELISPOT—Day 3	0.278	0.008
CD27+/Transitional B Cells (% of B Cells)—Day 3	B-Cell ELISPOT—Day 3	0.304	0.003
CD27+/Transitional B Cells (% of B Cells)—Day 28	B-Cell ELISPOT—Day 28	0.262	0.011
CD27+/Transitional B Cells (% of B Cells)—Day 75	B-Cell ELISPOT—Day 75	0.248	0.015
CD27+/Transitional B Cells (% of B Cells)—Day 3	B-Cell ELISPOT—(Day 3—Day 0)	0.235	0.025
CD27+/Transitional B Cells (% of B Cells)—(Day 3—Day 0)	B-Cell ELISPOT—(Day 3—Day 0)	0.218	0.04
IgD+CD27- B Cells (% of B Cells)—Day 0	B-Cell ELISPOT—(Day 75—Day 0)	-0.223	0.034
IgD+CD27- B Cells (% of B Cells)—Day 3	B-Cell ELISPOT—Day 3	-0.253	0.016
IgD+CD27- B Cells (% of B Cells)—Day 0	B-Cell ELISPOT—Day 75	-0.214	0.04
IgD+CD27- B Cells (% of B Cells)—Day 3	B-Cell ELISPOT—Day 75	-0.252	0.016
IgD+CD27- B Cells (% of B Cells)—Day 28	B-Cell ELISPOT—(Day 28—Day 0)	-0.227	0.028
IgD+CD27- B Cells (% of B Cells)—Day 28	B-Cell ELISPOT—(Day 75—Day 0)	-0.253	0.014
IgD+CD27-/Memory B Cells (% of B Cells)—Day 3	B-Cell Elispot—Day 3	-0.308	0.003
IgD+CD27-/Memory B Cells (% of B Cells)—Day 0	B-Cell Elispot—Day 75	-0.237	0.024
IgD+CD27-/Memory B Cells (% of B Cells)—Day 3	B-Cell Elispot—Day 75	-0.259	0.013
IgD+CD27-/Memory B Cells (% of B Cells)—Day 28	B-Cell ELISPOT—(Day 28—Day 0)	-0.216	0.037
IgD+CD27-/Memory B Cells (% of B Cells)—Day 28	B-Cell ELISPOT—(Day 75—Day 0)	-0.250	0.015
IgD+CD27-/Memory B Cells (% of B Cells)—Day 0	B-Cell ELISPOT—(Day 75—Day 0)	-0.218	0.038
IgD+CD27-/Naive B Cells (% of B Cells)–(Day 3—Day 0)	B-Cell ELISPOT—(Day 3—Day 0)	-0.219	0.039
IgD+CD27-/Transitional B Cells (% of B Cells)—Day 3	B-Cell ELISPOT—(Day 3—Day 0)	0.239	0.022
IgD-CD27-/Naive B Cells (% of B Cells)—Day 3	B-Cell ELISPOT—Day 28	0.235	0.025
IgD-CD27-/Naive B Cells (% of B Cells)—Day 3	B-Cell ELISPOT—Day 75	0.233	0.027
IgD-CD27-/Naive B Cells (% of B Cells)—Day 75	B-Cell ELISPOT—Day 75	0.302	0.003
IgD-CD27-/Naive B Cells (% of B Cells)—Day 3	B-Cell ELISPOT—(Day 28—Day 0)	0.219	0.037
CD20-/CD27highCD38high Plasma Cells (% of B Cells)—Day 75	B-Cell ELISPOT—Day 75	0.225	0.027

^a^Spearman’s rank r

^b^Only correlations with p-values below or equal to 0.05 are presented

^c^Influenza A/H1N1-specific memory-like IgG B cell ELISPOT response

### Multivariable Analysis of Early (Day 3) Variables/Factors Influencing Peak (Day 28) Influenza-Specific Humoral Immune Response after Vaccination

**Fig 3:**
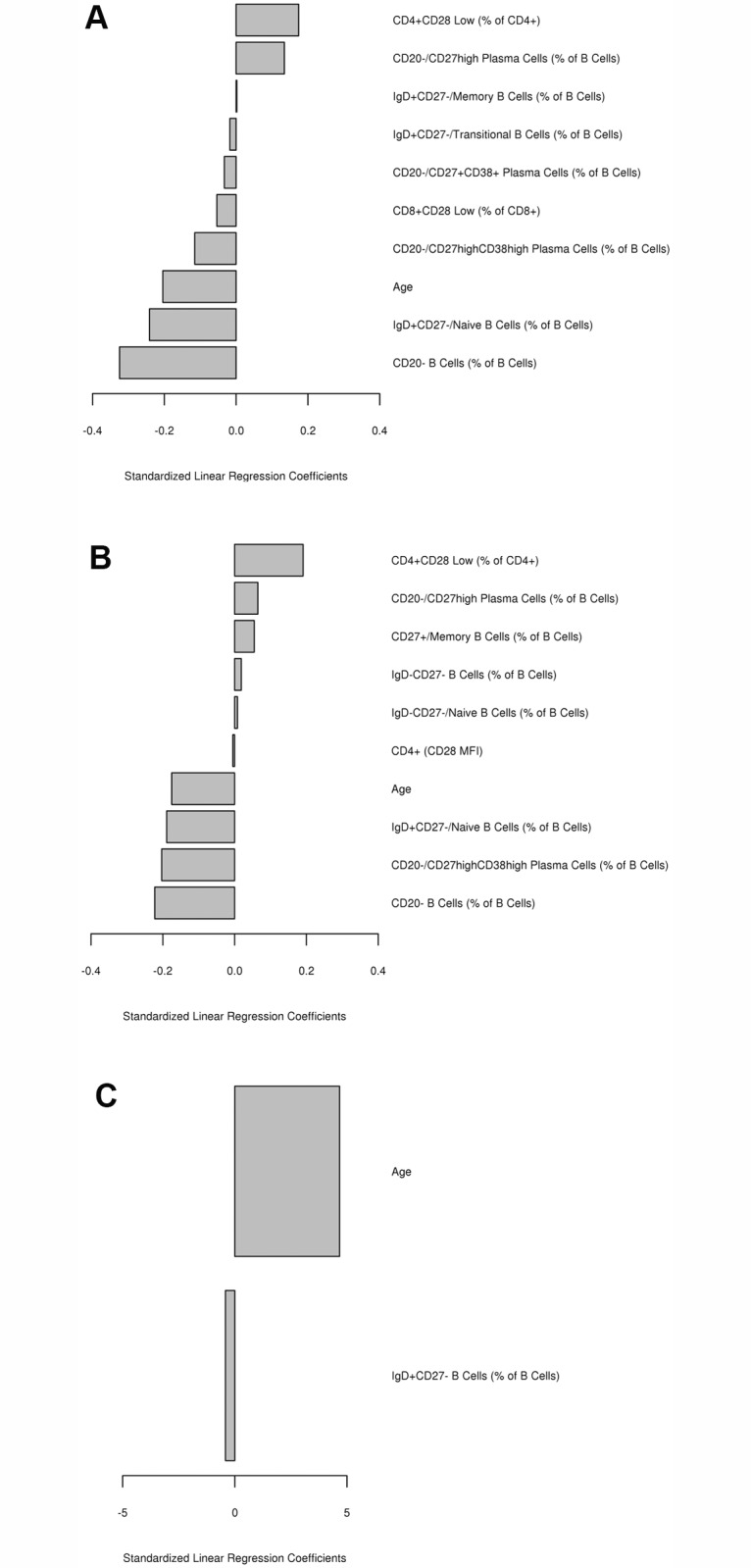
Multivariable associations of early variables with peak influenza/H1N1 vaccine-induced immune responses. Standardized linear regression coefficients from the elastic net penalized linear regression models for association of early (Day 3) variables with peak (Day 28) influenza-specific humoral immune response outcomes (HAI, VNA, B cell ELISPOT). Standardized linear regression coefficients for early variables associated with: **A.** Day 28 HAI response; **B**. Day 28 VNA response; and **C**. Day 28 B cell ELISPOT response. The model with the minimum cross validated mean squared error is presented.

The multivariable analysis demonstrated that age, percentage of CD8+CD28low T cells, IgD+CD27- naïve B cells, and percentage overall CD20- B cells and CD20-CD27highCD38high plasma cells/plasmablasts, all measured at Day 3 post-vaccination, were negatively associated with the peak Day 28 HAI antibody response (r^2^ = 0.31 for the model) and/or peak Day 28 VNA antibody response (r^2^ = 0.30 for the model), as illustrated in Fig. 3 (3a and 3b). The percentage CD4+CD28low T cells, CD20-CD27high plasma cells, and percentage memory B cells (measured at Day 3) displayed positive associations with HAI/VNA Day 28 antibody titer (Fig. 3). Age was positively associated with the observed Day 28 memory B cell ELISPOT response (r^2^ = 0.06 for the model, Fig. 3c).

## Discussion

Numerous data from the literature, including our own, point to age-related perturbations observed in all compartments of the immune system including both innate and adaptive humoral and cellular immune functions. The hallmarks of immunosenescence are low-grade inflammation, diminished ability to respond to foreign antigens, as well as increased susceptibility to infections, increased autoimmune responses, and compromised immunological memory [6]. Some of the major age-related functional deficiencies linked to immunosenescence include: defects in dendritic cell, macrophage and NK cell populations; defects in pattern recognition receptors and their function; defects in antigen presentation; myeloid-biased hematopoietic cell differentiation; thymic involution and contraction of the T cell repertoire; T cell CD28 and CD27 loss and gain in negative regulatory receptors; raise in anergic CD28-/low T cells and T regulatory T cells (Treg); reduced TCR diversity; reduced and/or altered T cell activation (e.g., increased DUSP4 transcription in activated CD4+ T cells with negative effect on influenza-specific B cell expansion and antibody response), signaling and functionality; altered TCR sensitivity (as a result of loss of miR-181a and increased DUSP6 in naive CD4+ T cells); disturbances in cytokine and chemokine secretion; latent/persistent infections (in particular, CMV) leading to increase of terminally differentiated effector CD8+ T cells and T cell exhaustion; impaired T cell help to B cells, diminished B cell repertoire (in size and diversity) with oligoclonal expansion; and perturbed B cell activation and effector function (e.g., downregulation of the transcription factor E47, and reduction of activation-induced cytidine deaminase/AID, which induces class switch recombination and somatic hypermutation), leading to impaired immune response with reduced antibody specificity and affinity [6,10,11,28,29,30,31,32,33,34,35,36,37,38,39,40,41].

Although it is largely accepted that age and age-related immunological perturbations have a large impact on immune response after vaccination in general, and influenza vaccination in particular, a modicum of data is available in humans regarding associations between specific quantitative markers of immunosenescence and immunity to influenza vaccine [6,7,8,9,10,42,43]. Therefore, in this study, we examined and identified associations between well-established immunosenescence markers (and age) and variations in longitudinal humoral immune profiles following seasonal influenza vaccination in 106 older individuals.

Measuring influenza vaccine-induced humoral immunity by HAI is considered the gold standard in influenza serology because it provides a correlate of protection (any HAI titer ≥1:40) that predicts vaccine efficacy against influenza virus in healthy adults and children [44]. However, HAI titer appears to be less applicable as a correlate of protection in high-risk populations (e.g., older adults) for developing severe influenza, and alternative markers of humoral (neutralizing antibodies, influenza virus-specific B cell ELISPOT response) and/or cellular immunity (granzyme B levels) have been suggested to more efficiently measure vaccine response and/or complement HAI response to better predict vaccine efficacy in this age group [18,19,42,44,45].

Consistent with other reports, the results from our current study clearly demonstrated the expected dynamics of the influenza vaccine-induced humoral immunity, with a peak of influenza A/H1N1-specific HAI and neutralization titers observed at Day 28 post-vaccination and a high correlation between the HAI and neutralizing antibody response [46,47]. Although 99% (105 out of 106) of the individuals had seroprotective antibody levels (HAI titer ≥1:40) at Day 28 and Day 75, the observed seroconversion rate of 39% in our study of older and elderly healthy subjects (≥4-fold increase in HAI titer from Day 0 to Day 28 post-vaccination with a non-adjuvanted influenza A/H1N1-containing vaccine, which is a metric used by the FDA and the World Health Organization for vaccine evaluation) was relatively low, but comparable to other studies of older individuals (21–76%), and achieving the threshold for influenza vaccine seroconversion (>30%) established for vaccine licensure [48,49,50,51]. Even though comparisons of immunogenicity results between different vaccine studies are confounded by differences in study design, vaccine formulation, study population and data analysis, reports from the literature and our current findings confirm the reduced immune response to influenza vaccination in older and elderly individuals [4,5,6,7,48,49,50,51]. Influenza A/H1N1vaccine-specific effector B cells (predominantly isotype-switched IgG antibody-secreting cells/ASCs, plasmablasts) are differentiated from stimulated naïve and memory B cells; they transiently peak 5–10 days following influenza vaccination, after which they quickly subside to low, hardly detectable levels (usually by day 14 after vaccination), and are shown to correlate with antibody titers [13,14,47]. Our study was not designed to study ASCs/plasmablasts’ kinetics and since we did not have the appropriate time points (post-vaccination blood draw at Day 5–10), it is not surprising that we did not detect any measurable changes in influenza A/H1N1-specific ASCs/plasmablasts’ concentrations. We were, however, able to monitor the pre-/post-vaccination kinetics of influenza virus-positive memory-like IgG B cell frequencies (as measured by ELISPOT after *in vitro* polyclonal stimulation and differentiation into ASCs), and their correlation with antibody response and immunosenescence markers.

The major goal of vaccination is to generate a recall and/or induction of immunological memory and provide long-term protection against disease upon subsequent wild type virus exposure [47]. Memory B cells are responsible for driving the rapid anamnestic antibody response and also play a role in refilling/sustaining the pool of long-lived plasma cells [24]. Although quantitatively assessed after influenza vaccination, this important immune cell population has not been correlated with antibody response in the context of immunosenescence in older individuals [52]. Notably, our data provides evidence for a positive correlation between the early change in influenza virus-specific memory-like IgG B cell ELSPOT response (Day 3 compared to baseline), and the observed increase in both influenza A/H1N1-specific HAI and neutralizing antibodies at the peak (Day 28) and the “return to homeostasis” (Day 75) immune response timepoints. In addition, we found multiple correlations between the overall (non-specific) CD20+/CD27+/B cell-, CD27+ /memory B cell-, and IgD-CD27-/memory B cell-frequencies and influenza virus-specific antibody titers and/or memory-like IgG B cell ELSPOT response. This confirms, and is consistent with, previous observations that the immune response to influenza A/H1N1 vaccine is predominantly a recall response, arising from pre-existing cross-reactive memory B cells, and favors a humoral immune response with broad neutralizing activity and antibodies directed against epitopes in the globular head, as well as the stem region (more rare) of the viral hemagglutinin [53,54]. Global analysis of B cell repertoire after influenza vaccination in the elderly has also demonstrated reduced B cell clonal diversity with an increased prevaccination mutation content due to memory B cells with a higher baseline somatic hypermutation [55]. The kinetics of other immune response (flow cytometry) variables relative to vaccination (e.g., overall IgD+CD27- B cells, IgD+CD27-/naive B cells, IgD+CD27-/transitional B cells, etc.), and the observed correlations between these variables and influenza vaccine-specific immune outcomes in our study, suggest the involvement of other B cell populations and immune response mechanisms (e.g., *de novo* antibody response) in the overall response after immunization in older individuals.

Previous reports from the literature have linked the decreased humoral and cellular immune response after influenza vaccination to advanced age and/or markers of immunological aging[6,7,9,10,18,56,57,58]. As expected, our univariable and multivariable analysis of influenza vaccine-induced immune responses in older and elderly healthy individuals (50 to 74 years old) found a weak negative correlation between age and the peak antibody response (both HAI and VNA) at day 28 following influenza vaccination (but not the memory B cell ELISPOT response). Since chronological age does not necessarily correlate with immunological aging, we assessed the relationships between established markers of immunosenescence (mostly T cell-based) and all influenza vaccine-specific longitudinal humoral (or related to humoral immunity) outcomes. The measured TRECs (extra-chromosomal DNA byproducts of T-cell receptor rearrangement, expressed in T cells of thymic origin) are an important indicator of thymic output and function, numerical T-cell competence and TCR recombination, all decreasing with age, but this biomarker of immunosenescence has never been assessed for correlations with vaccine response in healthy humans [59]. In our study, TREC levels exhibited significant correlations with only with the baseline and early post-vaccination influenza A/H1N1-specific memory IgG B cell ELSPOT frequencies, as well as with the overall IgD-/CD27- memory B cell frequencies. This is in concert with animal studies demonstrating that IL-7-treated old macaques with increased TREC levels responded better to inactivated influenza vaccine, with stronger influenza antigen-specific proliferative response and higher HAI titers [60]. Telomerase activity (associated with increased TERT mRNA) compensates for telomere loss during cell division/proliferation and is intrinsically linked to replicative senescence, T lymphocyte development and activation, maintenance of the immune repertoire, CD28 expression on T cells, and immunological aging [6,9,10]. In addition, telomerase is induced during B-cell activation by stimulation of the immunoglobulin receptors and has been linked to B-cell function, maintenance/longevity of memory CD8+ T cell population induced by viral infection, and immune response to vaccination [9,10,61,62]. Consistent with the aforementioned reports from the literature, we observed multiple correlations between TERT mRNA expression and the overall frequencies of several B cell and plasma cell populations, as well as a positive correlation between early change in TERT expression and the increase in the influenza virus-specific B cell ELISPOT response at day 28 post-vaccination (compared to baseline). These observations confirm the fundamental importance of telomerase activity for cell proliferation, function and immune response after vaccination. Previous studies have demonstrated associations between the expression of the costimulatory receptor CD28 on CD8+ T cells (CD8+T cells with CD28- (null) expression/% of CD8+ T cells), but not on CD4+ T cells, with the defective humoral immune response (HAI titers) after influenza vaccination, Th1/Th2 cytokine disbalance and the development of immune deficiency in the elderly [6,7,8]. CD28 is a multi-faceted co-stimulatory molecule and its low expression is a marker of T cell senescence. CD28 function is important for the proper antigen-mediated T cell activation, T cell proliferation and survival, cytotoxicity and immunoregulation [63]. Our univariable analysis found consistent (although weak) correlations between the expression levels of CD28 on both CD8+ and CD4+ T cells and influenza vaccine-induced memory-like IgG B cell ELISPOT response measured at different timepoints, but not antibody titers. Importantly, our multivariable analysis of early Day 3 variables associated with peak Day 28 influenza-specific HAI and/or VNA antibody response demonstrated negative associations between the percentage of CD8+CD28low T cells (for HAI), as well as the percentage IgD+CD27- naïve B cells, percentage overall CD20- B cells and plasmablasts (at Day 3), and peak antibody titers. These findings are similar to other published reports, and provide solid evidence for the importance of immunosenescence and, in particular, CD28-associated T cell immune abnormalities in older individuals for the observed lower immune response after influenza vaccination in this age group [6,7,8]. In particular, our study adds to the existing knowledge by demonstrating the impact of costimulatory receptor CD28 expression on CD8+ and CD4+ T cells not only on the development of antibody response (antibody titers) following influenza vaccination, but also on influenza-specific memory B cell ELISPOT response. The knowledge gained may help to predict vaccine immunogenicity and/or open new avenues (e.g., functional restoration of senescence, CD28 expression and proliferation by IL-12 stimulation and/or natural p53 isoform [Δ133p53] induction) to achieve better vaccine response in older individuals [63,64,65].

The strengths of our study include the comprehensive evaluation of influenza vaccine-specific and overall humoral immune variables and their dynamics in the settings of a longitudinal study of older and elderly individuals (50–74 years old) immunized with influenza A/H1N1-containing vaccine. It is important to note that our study cohort included a segment of the population with an age range from when immunological aging is starting to emerge to more apparent age-related immunological deficiencies, and from when the risk for influenza-related complications (morbidity and mortality) is measurably starting to increase to a relatively high risk of influenza-related complications in the elderly (65+ years old). While this study design may mitigate some of the findings, we have structured our approach to take advantage of the opportunity to study immunosenescence, as well as the relatively healthy immune system in older adults, in the setting of immune response to influenza vaccination. We have also assessed a relatively large number of associations and chose to be liberal in discussing results significant at the 5% level (the per-test error rate) in our effort to be comprehensive in evaluating the role of age and immunosenescence on vaccine response. We report actual p-values for all univariable associations, allowing readers to apply a per-experiment multiple comparison penalty and to evaluate the level of evidence (in light of all other data presented) when interpreting the data. In addition, we also provide data from multivariable analysis in order to more thoroughly define the early variables/factors influencing peak (Day 28) influenza-specific immune responses after vaccination.

In conclusion, our study findings help to explain the reduced influenza vaccine responsiveness observed in older individuals and may shed light on the underlying biological mechanisms (e.g., CD28-associated T cell immune abnormalities and TERT expression)leading to variation in immune response to vaccination/infection. The knowledge gained may be used to elucidate the critical factors and parameters of immune response heterogeneity among the elderly, to develop more effective influenza vaccines and/or approaches for this age group.

## Supporting Information

S1 Table Distribution of humoral immune response variables over time in a cohort of 106 older individuals. ^a^Antibody titers are presented as the inverse of the greatest serum dilution that still gave a positive result. ^b^Influenza A/H1N1-specific memory-like IgG B cell counts by ELISPOT: SFUs per 2x10^5^ PBMCs.
(DOCX)

S2 Table Sex differences in immune response variables in influenza vaccine recipients. ^a^Results are presented as median (25%, 75% IQR). ^b^Wilcoxon rank sum test with continuity correction.
(DOCX)

S3 Table Significance in timepoint variation of humoral immune response variables. ^a^Wilcoxon signed rank test for between timepoints comparisons; Friedman rank sum test for the overall comparisons. Only statistically significant differences (below 0.05) are presented. ^b^Influenza A/H1N1-specific memory-like IgG B cell ELISPOT response.
(DOCX)
