# Supplementary material for: The Impact of Immunosenescence on Humoral Immune Response Variation after Influenza A/H1N1 Vaccination in Older Subjects
Source: PLoS One. 2015 Mar 27;10(3):e0122282. doi: 10.1371/journal.pone.0122282 (PMC4376784; doi:10.1371/journal.pone.0122282)
Supplement: S1 Table — aAntibody titers are presented as the inverse of the greatest serum dilution that still gave a positive result. bInfluenza A/H1N1-specific memory-like IgG B cell counts by ELISPOT: SFUs per 2x10^5^ PBMCs. (DOCX) [file pone.0122282.s001.docx]

**Supplemental Table 1**. Distribution of humoral immune response variables over time in a cohort of 106 older individuals

| **Immune variable** | **Time point** | **N** | **Min** | **25^th^%** | **Median** | **75^th^%** | **Max** |
| --- | --- | --- | --- | --- | --- | --- | --- |
| HAI^a^ | Overall | 424 | 5 | 80 | 160 | 320 | 2560 |
| HAI | Day 0 | 106 | 5 | 40 | 80 | 280 | 1280 |
| HAI | Day 3 | 106 | 5 | 80 | 80 | 160 | 2560 |
| HAI | Day 28 | 106 | 5 | 80 | 320 | 640 | 2560 |
| HAI | Day 75 | 106 | 5 | 80 | 320 | 320 | 2560 |
| B-Cell ELISPOT ^b^ | Overall | 424 | -8 | 6 | 17 | 36 | 227 |
| B-Cell ELISPOT | Day 0 | 106 | -2 | 5 | 11 | 22 | 83 |
| B-Cell ELISPOT | Day 3 | 106 | -8 | 3 | 8 | 20 | 81 |
| B-Cell ELISPOT | Day 28 | 106 | 0 | 16 | 36 | 60 | 209 |
| B-Cell ELISPOT | Day 75 | 106 | 0 | 11 | 23 | 38 | 227 |
| VNA^a^ | Overall | 424 | 5 | 80 | 160 | 320 | 2560 |
| VNA | Day 0 | 106 | 5 | 40 | 80 | 320 | 2560 |
| VNA | Day 3 | 106 | 5 | 80 | 160 | 320 | 2560 |
| VNA | Day 28 | 106 | 5 | 160 | 320 | 640 | 2560 |
| VNA | Day 75 | 106 | 5 | 160 | 320 | 640 | 2560 |
| CD20+ B Cells (% B Cells) | Overall | 373 | 1.61 | 7.03 | 10.01 | 14.26 | 52.88 |
| CD20+ B Cells (% B Cells) | Day 0 | 91 | 2.41 | 7.62 | 10.33 | 15.47 | 50.76 |
| CD20+ B Cells (% B Cells) | Day 3 | 91 | 1.61 | 6.68 | 8.74 | 13.27 | 34.86 |
| CD20+ B Cells (% B Cells) | Day 28 | 94 | 2.78 | 7.05 | 9.98 | 14.57 | 52.88 |
| CD20+ B Cells (% B Cells) | Day 75 | 97 | 2.68 | 6.58 | 10.23 | 13.83 | 37.38 |
| CD20+/CD27+ B Cells (% of B Cells) | Overall | 373 | 0.24 | 1.70 | 2.50 | 3.80 | 15.38 |
| CD20+/CD27+ B Cells (% of B Cells) | Day 0 | 91 | 0.41 | 1.89 | 2.66 | 3.87 | 15.38 |
| CD20+/CD27+ B Cells (% of B Cells) | Day 3 | 91 | 0.24 | 1.74 | 2.27 | 3.53 | 14.97 |
| CD20+/CD27+ B Cells (% of B Cells) | Day 28 | 94 | 0.44 | 1.70 | 2.48 | 4.27 | 11.32 |
| CD20+/CD27+ B Cells (% of B Cells) | Day 75 | 97 | 0.24 | 1.57 | 2.61 | 3.91 | 12.81 |
| CD27+/Memory B Cells (% of B Cells) | Overall | 373 | 0.15 | 0.89 | 1.50 | 2.30 | 11.81 |
| CD27+/Memory B Cells (% of B Cells) | Day 0 | 91 | 0.20 | 0.94 | 1.57 | 2.33 | 9.48 |
| CD27+/Memory B Cells (% of B Cells) | Day 3 | 91 | 0.15 | 0.78 | 1.36 | 2.01 | 11.81 |
| CD27+/Memory B Cells (% of B Cells) | Day 28 | 94 | 0.25 | 0.93 | 1.54 | 2.56 | 8.43 |
| CD27+/Memory B Cells (% of B Cells) | Day 75 | 97 | 0.16 | 0.96 | 1.48 | 2.26 | 8.13 |
| CD27+/Naive B Cells (% of B Cells) | Overall | 373 | <0.01 | 0.08 | 0.13 | 0.23 | 9.69 |
| CD27+/Naive B Cells (% of B Cells) | Day 0 | 91 | <0.01 | 0.09 | 0.14 | 0.24 | 5.20 |
| CD27+/Naive B Cells (% of B Cells) | Day 3 | 91 | 0.01 | 0.07 | 0.11 | 0.21 | 9.69 |
| CD27+/Naive B Cells (% of B Cells) | Day 28 | 94 | <0.01 | 0.09 | 0.15 | 0.22 | 5.10 |
| CD27+/Naive B Cells (% of B Cells) | Day 75 | 97 | 0.01 | 0.07 | 0.12 | 0.22 | 9.49 |
| CD27+/Transitional B Cells (% of B Cells) | Overall | 373 | 0.02 | 0.26 | 0.49 | 0.93 | 5.82 |
| CD27+/Transitional B Cells (% of B Cells) | Day 0 | 91 | 0.04 | 0.28 | 0.47 | 0.98 | 5.82 |
| CD27+/Transitional B Cells (% of B Cells) | Day 3 | 91 | 0.02 | 0.25 | 0.47 | 0.89 | 3.13 |
| CD27+/Transitional B Cells (% of B Cells) | Day 28 | 94 | 0.02 | 0.27 | 0.48 | 0.89 | 3.75 |
| CD27+/Transitional B Cells (% of B Cells) | Day 75 | 97 | 0.04 | 0.23 | 0.53 | 0.95 | 4.59 |
| IgD+CD27- B Cells (% of B Cells) | Overall | 373 | 0.48 | 3.13 | 5.24 | 7.91 | 30.45 |
| IgD+CD27- B Cells (% of B Cells) | Day 0 | 91 | 0.48 | 3.54 | 5.43 | 8.12 | 30.45 |
| IgD+CD27- B Cells (% of B Cells) | Day 3 | 91 | 0.68 | 3.08 | 4.84 | 6.81 | 20.80 |
| IgD+CD27- B Cells (% of B Cells) | Day 28 | 94 | 0.64 | 3.11 | 5.03 | 7.73 | 29.41 |
| IgD+CD27- B Cells (% of B Cells) | Day 75 | 97 | 0.84 | 3.00 | 5.47 | 8.52 | 20.62 |
| IgD+CD27-/Memory B Cells (% of B Cells) | Overall | 373 | 0.34 | 2.20 | 3.60 | 5.79 | 28.14 |
| IgD+CD27-/Memory B Cells (% of B Cells) | Day 0 | 91 | 0.34 | 2.31 | 3.65 | 6.42 | 26.83 |
| IgD+CD27-/Memory B Cells (% of B Cells) | Day 3 | 91 | 0.58 | 1.83 | 3.28 | 4.96 | 18.08 |
| IgD+CD27-/Memory B Cells (% of B Cells) | Day 28 | 94 | 0.45 | 1.94 | 3.52 | 5.46 | 28.14 |
| IgD+CD27-/Memory B Cells (% of B Cells) | Day 75 | 97 | 0.64 | 2.23 | 3.78 | 6.11 | 18.02 |
| IgD+CD27-/Naive B Cells (% of B Cells) | Overall | 373 | <0.01 | 0.06 | 0.11 | 0.22 | 1.53 |
| IgD+CD27-/Naive B Cells (% of B Cells) | Day 0 | 91 | 0.01 | 0.06 | 0.13 | 0.25 | 1.53 |
| IgD+CD27-/Naive B Cells (% of B Cells) | Day 3 | 91 | <0.01 | 0.05 | 0.09 | 0.17 | 0.89 |
| IgD+CD27-/Naive B Cells (% of B Cells) | Day 28 | 94 | 0.01 | 0.06 | 0.10 | 0.23 | 1.02 |
| IgD+CD27-/Naive B Cells (% of B Cells) | Day 75 | 97 | <0.01 | 0.05 | 0.11 | 0.20 | 1.42 |
| IgD+CD27-/Transitional B Cells (% of B Cells) | Overall | 373 | 0.06 | 0.50 | 1.10 | 1.83 | 10.52 |
| IgD+CD27-/Transitional B Cells (% of B Cells) | Day 0 | 91 | 0.07 | 0.51 | 1.12 | 1.99 | 10.52 |
| IgD+CD27-/Transitional B Cells (% of B Cells) | Day 3 | 91 | 0.06 | 0.48 | 0.98 | 1.75 | 8.49 |
| IgD+CD27-/Transitional B Cells (% of B Cells) | Day 28 | 94 | 0.09 | 0.52 | 1.15 | 1.74 | 6.49 |
| IgD+CD27-/Transitional B Cells (% of B Cells) | Day 75 | 97 | 0.08 | 0.53 | 1.11 | 1.84 | 6.62 |
| IgD-CD27- B Cells (% of B Cells) | Overall | 373 | 0.25 | 0.86 | 1.35 | 2.15 | 39.77 |
| IgD-CD27- B Cells (% of B Cells) | Day 0 | 91 | 0.30 | 0.99 | 1.68 | 2.54 | 39.77 |
| IgD-CD27- B Cells (% of B Cells) | Day 3 | 91 | 0.28 | 0.76 | 1.35 | 2.33 | 18.97 |
| IgD-CD27- B Cells (% of B Cells) | Day 28 | 94 | 0.28 | 0.89 | 1.33 | 2.05 | 27.14 |
| IgD-CD27- B Cells (% of B Cells) | Day 75 | 97 | 0.25 | 0.83 | 1.24 | 1.79 | 15.03 |
| IgD-CD27-/Memory B Cells (% of B Cells) | Overall | 373 | 0.12 | 0.52 | 0.88 | 1.54 | 13.27 |
| IgD-CD27-/Memory B Cells (% of B Cells) | Day 0 | 91 | 0.17 | 0.65 | 1.08 | 1.68 | 7.31 |
| IgD-CD27-/Memory B Cells (% of B Cells) | Day 3 | 91 | 0.12 | 0.47 | 0.83 | 1.67 | 8.93 |
| IgD-CD27-/Memory B Cells (% of B Cells) | Day 28 | 94 | 0.14 | 0.57 | 0.84 | 1.39 | 13.27 |
| IgD-CD27-/Memory B Cells (% of B Cells) | Day 75 | 97 | 0.14 | 0.49 | 0.81 | 1.30 | 11.18 |
| IgD-CD27-/Naive B Cells (% of B Cells) | Overall | 373 | 0.02 | 0.12 | 0.18 | 0.33 | 33.38 |
| IgD-CD27-/Naive B Cells (% of B Cells) | Day 0 | 91 | 0.02 | 0.12 | 0.22 | 0.33 | 33.38 |
| IgD-CD27-/Naive B Cells (% of B Cells) | Day 3 | 91 | 0.03 | 0.11 | 0.17 | 0.30 | 16.18 |
| IgD-CD27-/Naive B Cells (% of B Cells) | Day 28 | 94 | 0.02 | 0.13 | 0.18 | 0.39 | 23.42 |
| IgD-CD27-/Naive B Cells (% of B Cells) | Day 75 | 97 | 0.02 | 0.11 | 0.17 | 0.33 | 12.94 |
| IgD-CD27-/Transitional B Cells (% of B Cells) | Overall | 373 | <0.001 | 0.05 | 0.13 | 0.23 | 2.93 |
| IgD-CD27-/Transitional B Cells (% of B Cells) | Day 0 | 91 | <0.001 | 0.06 | 0.15 | 0.27 | 2.93 |
| IgD-CD27-/Transitional B Cells (% of B Cells) | Day 3 | 91 | 0.005 | 0.05 | 0.13 | 0.22 | 1.97 |
| IgD-CD27-/Transitional B Cells (% of B Cells) | Day 28 | 94 | <0.001 | 0.06 | 0.14 | 0.22 | 2.54 |
| IgD-CD27-/Transitional B Cells (% of B Cells) | Day 75 | 97 | 0.006 | 0.05 | 0.11 | 0.22 | 2.11 |
| CD20- B Cells (% of B Cells) | Overall | 373 | 43.18 | 84.47 | 88.92 | 91.93 | 97.02 |
| CD20- B Cells (% of B Cells) | Day 0 | 91 | 44.79 | 82.53 | 88.05 | 91.19 | 96.78 |
| CD20- B Cells (% of B Cells) | Day 3 | 91 | 64.42 | 85.42 | 89.39 | 92.05 | 97.02 |
| CD20- B Cells (% of B Cells) | Day 28 | 94 | 43.18 | 84.07 | 89.19 | 92.20 | 96.67 |
| CD20- B Cells (% of B Cells) | Day 75 | 97 | 61.20 | 85.27 | 88.80 | 92.43 | 96.47 |
| CD20-/CD27+CD38+ Plasma Cells (% of B Cells) | Overall | 373 | 36.08 | 81.30 | 86.39 | 89.58 | 95.68 |
| CD20-/CD27+CD38+ Plasma Cells (% of B Cells) | Day 0 | 91 | 42.21 | 79.34 | 85.46 | 88.93 | 93.79 |
| CD20-/CD27+CD38+ Plasma Cells (% of B Cells) | Day 3 | 91 | 63.35 | 82.10 | 87.07 | 89.55 | 95.25 |
| CD20-/CD27+CD38+ Plasma Cells (% of B Cells) | Day 28 | 94 | 36.08 | 81.40 | 86.62 | 89.82 | 94.72 |
| CD20-/CD27+CD38+ Plasma Cells (% of B Cells) | Day 75 | 97 | 59.44 | 81.63 | 86.19 | 90.19 | 95.68 |
| CD20-/CD27high Plasma Cells (% of B Cells) | Overall | 373 | 0.12 | 0.70 | 1.35 | 2.33 | 6.74 |
| CD20-/CD27high Plasma Cells (% of B Cells) | Day 0 | 91 | 0.12 | 0.80 | 1.34 | 2.29 | 5.09 |
| CD20-/CD27high Plasma Cells (% of B Cells) | Day 3 | 91 | 0.16 | 0.70 | 1.44 | 2.30 | 5.74 |
| CD20-/CD27high Plasma Cells (% of B Cells) | Day 28 | 94 | 0.23 | 0.73 | 1.33 | 2.14 | 5.61 |
| CD20-/CD27high Plasma Cells (% of B Cells) | Day 75 | 97 | 0.22 | 0.68 | 1.35 | 2.33 | 6.74 |
| CD20-/CD27highCD38high Plasma Cells (% of B Cells) | Overall | 373 | <0.001 | 0.07 | 0.16 | 0.34 | 7.44 |
| CD20-/CD27highCD38high Plasma Cells (% of B Cells) | Day 0 | 91 | <0.001 | 0.07 | 0.16 | 0.40 | 7.22 |
| CD20-/CD27highCD38high Plasma Cells (% of B Cells) | Day 3 | 91 | <0.001 | 0.06 | 0.15 | 0.38 | 1.41 |
| CD20-/CD27highCD38high Plasma Cells (% of B Cells) | Day 28 | 94 | 0.005 | 0.07 | 0.15 | 0.34 | 7.44 |
| CD20-/CD27highCD38high Plasma Cells (% of B Cells) | Day 75 | 97 | 0.007 | 0.08 | 0.16 | 0.26 | 6.54 |

^a^Antibody titers are presented as the inverse of the greatest serum dilution that still gave a positive result.

^b^Influenza A/H1N1-specific memory-like IgG B cell counts by ELISPOT: SFUs per 2x10^5^ PBMCs
