# Supplementary material for: The Impact of Immunosenescence on Humoral Immune Response Variation after Influenza A/H1N1 Vaccination in Older Subjects
Source: PLoS One. 2015 Mar 27;10(3):e0122282. doi: 10.1371/journal.pone.0122282 (PMC4376784; doi:10.1371/journal.pone.0122282)
Supplement: S2 Table — aResults are presented as median (25%, 75% IQR). bWilcoxon rank sum test with continuity correction. (DOCX) [file pone.0122282.s002.docx]

**Supplemental Table 2.** Sex differences in immune response variables in influenza vaccine recipients

| **Immune response variable** | **Timepoint** | **Females**^a^ | **Males**^a^ | **p-value**^b^ | **Note** |
| --- | --- | --- | --- | --- | --- |
| CD20+ B Cells (% B Cells) | Day 0 | 13.6 (8.4,19.9) | 10.0 (6.5,10.9) | 0.003 | F>M |
| CD20+ B Cells (% B Cells) | Day 75 | 11.6 (8.0,17.6) | 8.4 (5.5,11.7) | 0.003 | F>M |
| CD20- B Cells (% of B Cells) | Day 0 | 85.3 (79.0,90.7) | 89.1 (87.9,92.3) | 0.004 | M>F |
| CD20- B Cells (% of B Cells) | Day 75 | 87.7 (81.7,90.8) | 90.8 (86.9,94.0) | 0.005 | M>F |
| IgD+CD27- B Cells (% of B Cells) | Day 75 | 6.9 (3.4,9.2) | 4.0 (2.3,6.4) | 0.006 | F>M |
| IgD+CD27-/Memory B Cells (% of B Cells) | Day 75 | 4.9 (2.4,6.9) | 3.0 (1.7,4.2) | 0.006 | F>M |
| CD20-/CD27+CD38+ Plasma Cells (% of B Cells) | Day 0 | 83.5 (76.1,88.8) | 86.4 (84.5,90.0) | 0.007 | M>F |
| IgD+CD27-/Memory B Cells (% of B Cells) | Day 0 | 4.1 (2.9,8.8) | 2.8 (1.9,4.7) | 0.008 | F>M |
| CD20-/CD27+CD38+ Plasma Cells (% of B Cells) | Day 75 | 85.0 (80.4, 88.4) | 88.3 (82.7,92.1) | 0.009 | M>F |
| IgD-CD27- B Cells (% of B Cells) | Day 75 | 1.4 (0.9,2.5) | 1.0 (0.7,1.6) | 0.015 | F>M |
| IgD+CD27- B Cells (% of B Cells) | Day 0 | 6.0 (3.8,11.1) | 4.6 (3.1,6.5) | 0.020 | F>M |
| IgD+CD27-/Transitional B Cells (% of B Cells) | Day 75 | 1.4 (0.7,2.3) | 0.7 (0.5,1.3) | 0.023 | F>M |
| CD20+ B Cells (% B Cells) | Day 3 | 10.0 (7.4,15.4) | 7.4 (6.4, 10.2) | 0.030 | F>M |
| CD20- B Cells (% of B Cells) | Day 3 | 88.5 (83.4,91.4) | 91.3 (88.5,92.6) | 0.035 | M>F |
| Influenza A/H1N1-specific memory-like IgG B cell ELISPOT | Day 28 | 37.5 (19.0, 65.0) | 33.0 (10.5,53.0) | 0.056 | F>M |
| Influenza A/H1N1-specific memory-like IgG B cell ELISPOT | Day 3 | 10.0 (3.0,23.5) | 5.0 (1.5, 14.5) | 0.061 | F>M |
| Influenza A/H1N1-specific memory-like IgG B cell ELISPOT | Day 0 | 14.5 (5.5,23.5) | 8.5 (3.0, 19.0) | 0.087 | F>M |
| Influenza A/H1N1-specific memory-like IgG B cell ELISPOT | Day 75 | 26.0 (11.0,44.0) | 19.0 (10.0,30.0) | 0.185 | F>M |

^a^Results are presented as median (25%, 75% IQR)

^b^Wilcoxon rank sum test with continuity correction
