# Supplementary material for: The Impact of Immunosenescence on Humoral Immune Response Variation after Influenza A/H1N1 Vaccination in Older Subjects
Source: PLoS One. 2015 Mar 27;10(3):e0122282. doi: 10.1371/journal.pone.0122282 (PMC4376784; doi:10.1371/journal.pone.0122282)
Supplement: S3 Table — aWilcoxon signed rank test for between timepoints comparisons; Friedman rank sum test for the overall comparisons. Only statistically significant differences (below 0.05) are presented. bInfluenza A/H1N1-specific memory-like IgG B cell ELISPOT response. (DOCX) [file pone.0122282.s003.docx]

**Supplemental Table 3**. Significance in timepoint variation of humoral immune response variables

| **Humoral Immune Response Variable** | **Test** | **P-value**^a^ | **Note** |
| --- | --- | --- | --- |
| HAI | Overall | 1.64E^-28^ |  |
| HAI | Day 28 vs Day 0 | 1.12E^-11^ | ↑ |
| HAI | Day 28 vs Day 3 | 9.37E^-10^ | ↑ |
| HAI | Day 75 vs Day 0 | 3.3E^-09^ | ↑ |
| HAI | Day 75 vs Day 3 | 6.72E^-09^ | ↑ |
| HAI | Day 75 vs Day 28 | 0.003 | ↓ |
| B-Cell ELISPOT^b^ | Overall | 5.77E^-26^ |  |
| B-Cell ELISPOT | Day 3 vs Day 0 | 0.001 | ↓ |
| B-Cell ELISPOT | Day 28 vs Day 0 | 1.48E^-13^ | ↑ |
| B-Cell ELISPOT | Day 28 vs Day 3 | 2.04E^-15^ | ↑ |
| B-Cell ELISPOT | Day 75 vs Day 0 | 6.33E^-08^ | ↑ |
| B-Cell ELISPOT | Day 75 vs Day 3 | 7.82E^-12^ | ↑ |
| B-Cell ELISPOT | Day 75 vs Day 28 | 3.17E^-09^ | ↓ |
| VNA | Overall | 8.4E^-32^ |  |
| VNA | Day 28 vs Day 0 | 1.22E^-12^ | ↑ |
| VNA | Day 28 vs Day 3 | 3.93E^-11^ | ↑ |
| VNA | Day 75 vs Day 0 | 6.55E^-12^ | ↑ |
| VNA | Day 75 vs Day 3 | 3.13E^-11^ | ↑ |
| CD20+ B Cells (% B Cells) | Overall | 0.035 |  |
| CD20+ B Cells (% B Cells) | Day 3 vs Day 0 | 0.002 | ↓ |
| CD20+ B Cells (% B Cells) | Day 28 vs Day 3 | 0.046 | ↑ |
| CD20+ B Cells (% B Cells) | Day 75 vs Day 3 | 0.005 | ↑ |
| CD20+/CD27+ B Cells (% of B Cells) | Overall | 0.024 |  |
| CD20+/CD27+ B Cells (% of B Cells) | Day 28 vs Day 3 | 0.015 | ↑ |
| CD20+/CD27+ B Cells (% of B Cells) | Day 75 vs Day 28 | 0.049 | ↑ |
| CD27+/Memory B Cells (% of B Cells) | Overall | 0.002 |  |
| CD27+/Memory B Cells (% of B Cells) | Day 28 vs Day 3 | 0.0006 | ↑ |
| CD27+/Memory B Cells (% of B Cells) | Day 75 vs Day 28 | 0.021 | ↓ |
| IgD+CD27- B Cells (% of B Cells) | Overall | 0.036 |  |
| IgD+CD27- B Cells (% of B Cells) | Day 3 vs Day 0 | 0.006 | ↓ |
| IgD+CD27- B Cells (% of B Cells) | Day 75 vs Day 0 | 0.04 | ↑ |
| IgD+CD27-/Naive B Cells (% of B Cells) | Overall | 0.015 |  |
| IgD+CD27-/Naive B Cells (% of B Cells) | Day 3 vs Day 0 | 0.002 | ↓ |
| IgD+CD27-/Naive B Cells (% of B Cells) | Day 75 vs Day 0 | 0.003 | ↓ |
| IgD-CD27- B Cells (% of B Cells) | Overall | 2.6E^-05^ |  |
| IgD-CD27- B Cells (% of B Cells) | Day 3 vs Day 0 | 0.002 | ↓ |
| IgD-CD27- B Cells (% of B Cells) | Day 75 vs Day 0 | 9.3E^-05^ | ↓ |
| IgD-CD27- B Cells (% of B Cells) | Day 75 vs Day 28 | 0.004 | ↓ |
| IgD-CD27-/Memory B Cells (% of B Cells) | Overall | 0.0002 |  |
| IgD-CD27-/Memory B Cells (% of B Cells) | Day 3 vs Day 0 | 0.003 | ↓ |
| IgD-CD27-/Memory B Cells (% of B Cells) | Day 75 vs Day 0 | 9.82E^-05^ | ↓ |
| IgD-CD27-/Memory B Cells (% of B Cells) | Day 75 vs Day 28 | 0.005 | ↓ |
| CD20- B Cells (% of B Cells) | Day 3 vs Day 0 | 0.004 | ↑ |
| CD20- B Cells (% of B Cells) | Day 75 vs Day 0 | 0.003 | ↑ |
| CD20-/CD27+CD38+ Plasma Cells (% of B Cells) | Day 3 vs Day 0 | 0.006 | ↑ |
| CD20-/CD27+CD38+ Plasma Cells (% of B Cells) | Day 75 vs Day 0 | 0.007 | ↑ |

^a^Wilcoxon signed rank test for between timepoints comparisons; Friedman rank sum test for the overall comparisons. Only statistically significant differences (below 0.05) are presented.

^b^Influenza A/H1N1-specific memory-like IgG B cell ELISPOT response
